# Phase Engineering of Nanogold: Non‐Close Packed Square Planes in A′B′ Stacking with a 0.5 Å Channel

**DOI:** 10.1002/adma.202517188

**Published:** 2025-12-16

**Authors:** Yitong Wang, Zhongyu Liu, Christopher G. Gianopoulos, Wei Zhang, Dominic Alfonso, Guiying He, Kristin Kirschbaum, Meng Zhou, Rongchao Jin

**Affiliations:** ^1^ Department of Chemistry Carnegie Mellon University Pittsburgh PA 15213 USA; ^2^ Department of Chemistry and Biochemistry University of Toledo Toledo OH 43606 USA; ^3^ Hefei National Research Center for Physical Sciences at the Microscale Department of Chemical Physics University of Science and Technology of China Hefei Anhui 230026 China; ^4^ National Energy Technology Laboratory United States Department of Energy Pittsburgh PA 15236 USA

**Keywords:** atomically precise gold nanoclusters, non‐fcc structure, phase engineering, ultrafast electron dynamics

## Abstract

Crystal phase engineering of inorganic materials is one of the most important strategies for achieving new material properties and functionality. Gold is well known to adopt the face‐centered‐cubic (fcc) structure, and it remains challenging to create new phases, but recent research on nanogold has provided exciting opportunities by achieving 2H (note: H denotes hexagonal), 4H, and body‐centered‐tetragonal (bct) phases. In the current work, an unusual non‐compact phase in 40‐atom gold nanoclusters is reported, which is in contrast to the close‐packed isomer of fcc structure. The non‐compact isomer exhibits exclusive square planes in a layer‐by‐layer stacking, hence giving rise to a unique 0.5 Å channel, whereas the fcc isomer is composed of close‐packed planes with a closed structure (i.e., no 1D channel). The two Au_40_ isomers lead to a nearly two orders of magnitude difference in electronic excited‐state lifetime. The attainment of the new non‐compact phase in nanogold presents potential opportunities in terahertz emission owing to the strong electron‐vibration coupling, ion channeling and ion selection, artificial photosynthesis, and biocatalysis.

## Graphical abstract

Gold is well known to adopt the face‐centered‐cubic (fcc) structure. This work presents an unusual non‐close‐packed phase attained in a 40‐atom gold nanocluster, which is in contrast with the close‐packed fcc quasi‐isomer. The non‐compact structure exhibits exclusive square planes in a layer‐by‐layer stacking, hence, possessing a unique 0.5 Å channel. Its photoexcited state lifetime is nearly two orders of magnitude shorter than that of the fcc isomer.

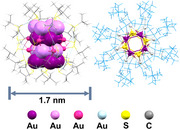

## 1 Introduction

Engineering the crystalline phase of materials is crucial for their functionality, as the physicochemical properties are dictated by the specific atomic packing structures.^[^ ^1^, ^2^, ^3^, ^4^ ^]^ However, it has remained challenging to engineer the crystalline phase of materials, such as the metallic vs semiconducting phase in 2D materials^[^ ^2^ ^]^ or Mo‐ and W‐based dichalcogenide nanocrystals.^[^ ^3^ ^]^ In the case of gold, bulk gold has a face‐centered cubic (fcc) structure,^[^ ^4^ ^]^ but nanogold can exhibit multiple new phases.^[^ ^4^, ^5^, ^6^, ^7^, ^8^ ^]^ Nanogold with the hexagonal close‐packing (hcp, also termed 2H) structure was reported by Zhang et al. in the synthesis of square nanosheets^[^ ^5^ ^]^ and also by Xia et al. in the synthesis of hexagonal nanostars mediated by fcc seeds.^[^ ^8^ ^]^ Other phases, including body‐centered tetragonal (bct)^[^ ^6^, ^9^, ^10^ ^]^ and body‐centered orthorhombic (bco) structures,^[^ ^6^ ^]^ as well as the 4H structure^[^ ^7^ ^]^ (i.e., close‐packed planes stacked in ABCB periodicity, where A to C represent close‐packed planes), have been attained. Deformation‐induced phase transformation of sub‐10 nm gold nanocrystals from the fcc to bct phase was studied by in situ transmission electron microscopy.^[^ ^9^ ^]^ These new phases of nanogold exhibit new properties such as electrocatalytic hydrogen evolution,^[^ ^3^ ^]^ optical and mechanical properties,^[^ ^8^, ^9^ ^]^ and thermodynamic behavior.^[^ ^6^, ^7^ ^]^ Recently, research on atomically precise gold nanoclusters (1–3 nm) has also obtained hcp and body‐centered cubic (bcc) structures,^[^ ^1^, ^8^, ^9^, ^10^, ^11^ ^]^ as well as mixed phases^[^ ^12^, ^13^ ^]^ in the cores of nanoclusters as determined by X‐ray crystallography. With such new phases, versatile functionality such as the structure‐dependent periodic evolution of electronic structure, near‐infrared photothermy and photoluminescence, catalytic activity in H_2_ evolution and CO_2_ reduction, as well as optoelectronic properties have been discovered.^[^ ^7^, ^8^, ^9^, ^10^, ^11^, ^12^, ^13^, ^14^, ^15^, ^16^ ^]^

Here, we report an unusual phase observed in a 40‐atom gold nanocluster formulated as Au_40_(S‐*t*Bu)_24_ (where S‐*t*Bu stands for *tert*‐butyl thiolate ligand). Unlike the fcc and hcp phases that are composed of close‐packed planes in ABC and AB periodicities, respectively,^[^ ^1^, ^7^ ^]^ the Au_40_ structure lacks the commonly observed close‐packed planes (denoted A, B, C) but possesses non‐compact square planes (denoted A′, B′) which are stacked in an A′B′A′B′ sequence. This structure escapes from the common icosahedral structures.^[^ ^4^, ^17^, ^18^, ^19^, ^20^ ^]^ In addition, the Au_40_(S‐*t*Bu)_24_ structure contrasts with a quasi‐isomer, i.e., the fcc Au_40_(*o*‐MBT)_24_ (where *o*‐MBT refers to *ortho*‐methylbenzenethiolate). The non‐compact core of Au_40_(S‐*t*Bu)_24_ leads to a 0.5 Å channel. Spectroscopic studies reveal that Au_40_(S‐*t*Bu)_24_ exhibits a short excited‐state lifetime (7.7 ns), while the Au_40_(*o*‐MBT)_24_ quasi‐isomer possesses a ≈640 ns lifetime; thus, the phase control of Au_40_ nanoclusters leads to a nearly two orders of magnitude difference in excited‐state lifetime. The non‐compact core of Au_40_(S‐*t*Bu)_24_ with a 1D channel holds promise in proton channeling, energy, and bio‐catalysis.

## 2 Results and Discussion

The Au_40_(S‐*t*Bu)_24_ nanocluster was synthesized via mild reduction of [Au(I)(S‐*t*Bu)]*_x_* precursor using a borane *tert*‐butylamine complex (i.e., weak reducing agent) or a controlled addition of NaBH_4_, followed by the purification of the crude product by thin‐layer chromatography (see Supporting Information for details of the synthesis and workup). Single crystals of Au_40_(S‐*t*Bu)_24_ were grown via liquid‐phase diffusion of methanol into a toluene solution of Au_40_(S‐*t*Bu)_24_ (methanol/toluene = 5:2 vol.). Single‐crystal X‐ray diffraction (SCXRD) analysis reveals that the Au_40_(S‐*t*Bu)_24_ nanoclusters are packed into a tetragonal P42(1)2 space group (Table S1, Supporting Information). Two identical configurations were identified, with a 78% possibility for the major site and 22% for the minor site (Figure S1, Supporting Information). Nevertheless, the discussions in the following text apply to both cases, as the structural differences between the two configurations are trivial.

**Figure 1:**
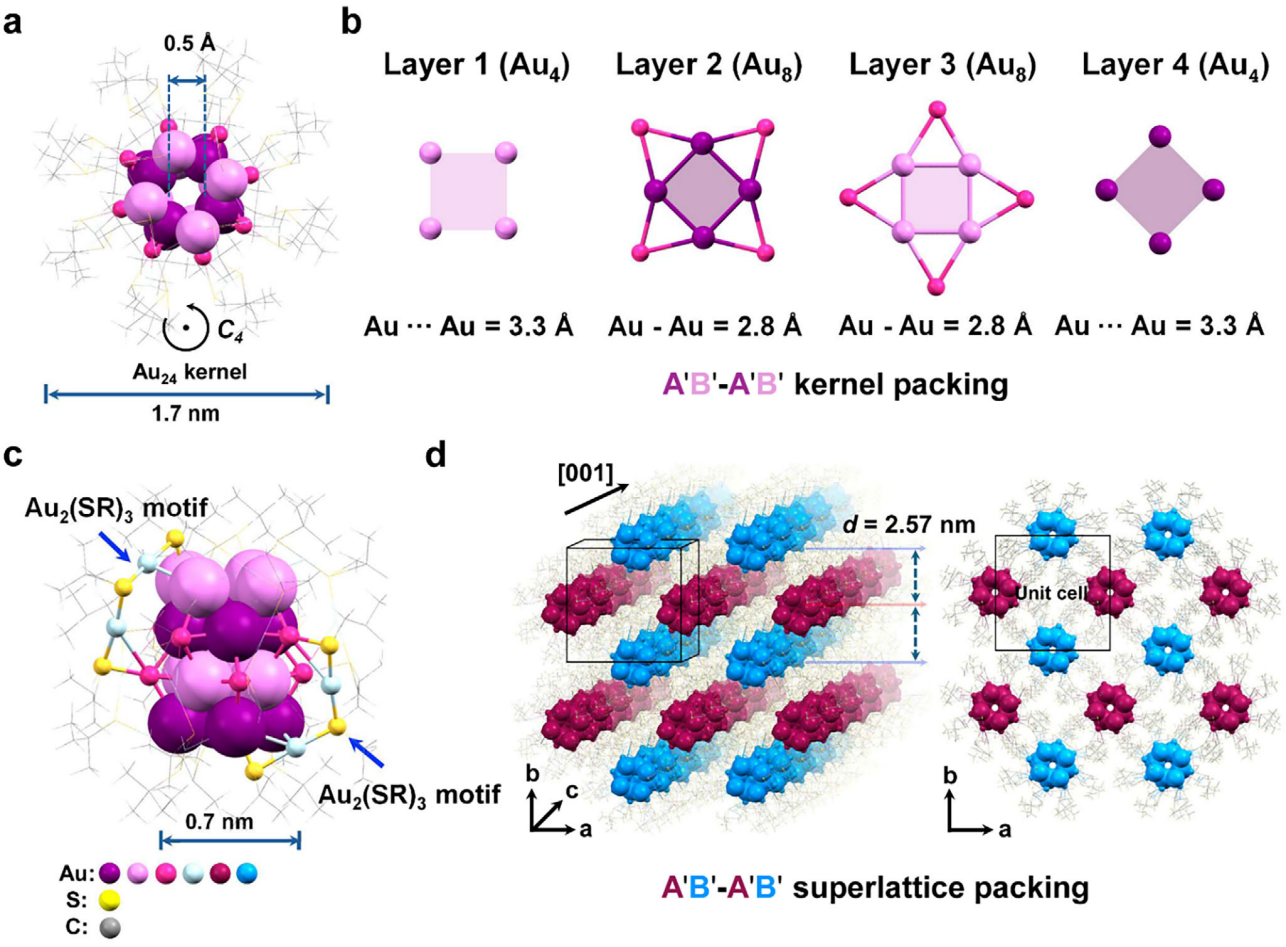
Anatomy of the X‐ray crystallographic structure of Au_40_(S‐*t*Bu)_24_. a) Total structure, b) individual layers of the Au_24_ kernel, c) protection of the Au_24_ kernel by dimeric staple‐like motifs (only two are shown for clarity, 8 total), and d) the superlattice structure.

The Au_40_(S‐*t*Bu)_24_ nanocluster is a slightly prolate core–shell structure, with the gold atoms packed in an unusual configuration (**Figure** **1**a), rather than the commonly observed icosahedral structure.^[^ ^17^, ^18^, ^19^ ^]^ An inner core of Au_24_ can be identified, which comprises four non‐close packed atomic layers of square shape (Figure 1b). The square planes are arranged in a staggered configuration, i.e., Au_4_‐Au_8_‐Au_8_‐Au_4_. Layers 1 and 4 squares have an edge length of 3.310 ± 0.003 Å and 3.303 ± 0.003 Å, whereas layers 2 and 3 exhibit a quadrangular star‐shaped structure. For layers 2 and 3, the inner Au_4_ square has an edge length of 2.839 ± 0.002 Å and 2.844 ± 0.002 Å. The interlayer distances from 1 to 4 are 2.257, 2.272, and 2.256 Å, which are shorter than the typical distance between atomic planes (2.35 Å) in bulk Au, leading to a more compact interlayer packing than the one in bulk Au (although not a denser packing). The centers of the four layers are perfectly aligned, forming a tunnel (0.5 Å diameter) through the nanocluster (Figure S1, Supporting Information), with the top and bottom Au_4_ squares being somewhat more open (square edge length ∼3.305 Å) than the middle two layers (square edge length ∼2.841 Å), hence, a channel. This feature contrasts with the typical nanoclusters,^[^ ^1^ ^]^ which were reported to be closed structures without any open channels. Although the previously reported Au_144_(SR)_60_ has a hollow 12‐atom icosahedron,^[^ ^19^ ^]^ it is not a planar layer‐by‐layer structure; hence, no open channel connecting the interior and exterior environments. The unique 1D channel in Au_40_(S‐*t*Bu)_24_ may present future opportunities for ion channeling (e.g., H^+^), ion selection, and new host‐guest chemistry, as the selective entering of H^+^ is quite important in energy and bio‐catalysis. The Au_24_ core possesses a four‐fold rotation axis (*C*_4_). As shown in Figure 1c, along the *C*_4_ axis, four Au_2_(SR)_3_ staple‐like motifs connect layer 1 and layer 3 of the kernel, and another four Au_2_(SR)_3_ staples connect layer 2 and layer 4 in a similar manner, but they are rotated by 45° along the *C*_4_ axis with respect to the first four staples. When viewing from the top and bottom directions of the *C*_4_ axis, the four staples derived from layer 1 and layer 4 (Figure S2, Supporting Information) can be seen rotating clockwise and counterclockwise from the two poles; thus, the shell structure is achiral. As a result, the total structure belongs to the *S*_8_ point group. The Au_40_(S‐*t*Bu)_24_ nanoclusters are packed into a tetragonal superlattice in the macroscopic crystal (Figure 1d).

An fcc Au_40_(*o*‐MBT)_24_ (where, *o*‐MBT = *ortho*‐methylbenzenethiolate) was previously reported (Figure S3, Supporting Information).^[^ ^21^ ^]^ The two Au_40_ nanoclusters have the same Au_40_(SR)_24_ formula except different –R groups, hence, a pair of quasi‐isomers. The packing density of ligands is 8.75 ligands nm^−2^ for Au_40_(*o*‐MBT)_24_ and 5.56 ligands nm^−2^ for Au_40_(S‐*t*Bu)_24_, calculated based on the surface area of the gold core, which is caused by the steric hindrance of the ligands.^[^ ^22^, ^23^ ^]^ Compared to the Au_40_(*o*‐MBT)_24_, the use of a bulkier ligand for Au_40_(S‐*t*Bu)_24_ results in less dense packing of ligands on the surface of the core. Eight staple motifs in Au_40_(S‐*t*Bu)_24_ are bound to 16 surface gold atoms, leaving 8 gold atoms uncoordinated. In contrast, there were only 3 uncoordinated Au atoms on the core surface of Au_40_(*o*‐MBT)_24_ (Figure S3, Supporting Information). The surface difference leads to the different cores of the two Au_40_ nanoclusters, one with a close‐packed fcc structure, while the other with a non‐compact structure featuring a 1D channel. The new phase observed in Au_40_(S‐*t*Bu)_24_ also differs from the previous prediction of Au_40_(SR)_24_ structures,^[^ ^24^, ^25^ ^]^ hence, contributing to the development of new theoretical models.^[^ ^26^, ^27^ ^]^

**Figure 2:**
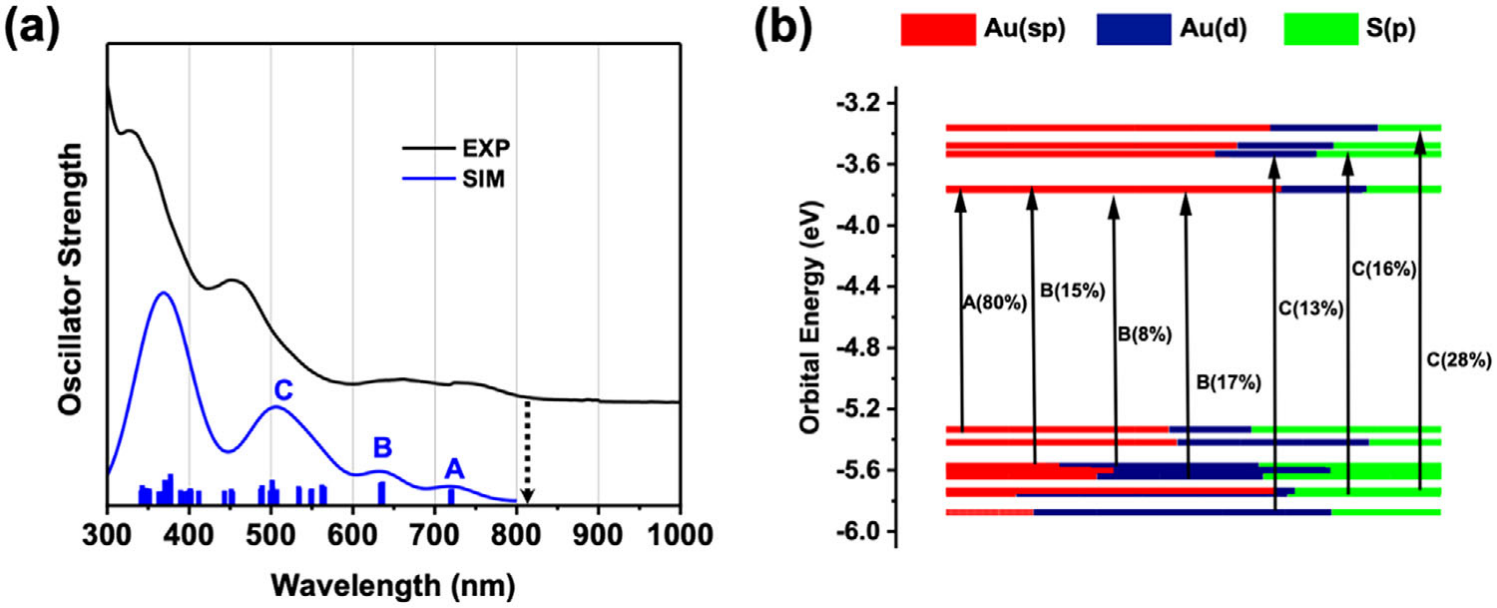
Optical and electronic properties of Au_40_(S‐*t*Bu)_24_. a) Experimental absorption spectrum of Au_40_(S‐*t*Bu)_24_ (black curve) and TDDFT‐simulated absorption spectrum of Au_40_(SCH_3_)_24_ (blue curve, the stick pattern indicates individual electronic transitions and their heights represent the oscillator strengths, b) Kohn–Sham (KS) orbital energy level diagram of Au_40_(SCH_3_)_24_, where the A, B and C transitions correspond to those marked in panel **a**, and the percentages in parentheses are the individual contribution of transitions (only predominant ones are shown) for the overall peak A, B or C. The atomic orbital contributions to KS orbitals are marked in colors.

The optical absorption spectrum of Au_40_(S‐*t*Bu)_24_ (dissolved in dichloromethane) is shown in **Figure** **2**a (solid line). An onset is observed at ≈800 nm (indicated by an arrow), hence, an optical gap of 1.55 eV, and peaks are observed at 736, 644, 460, and 336 nm. We conducted time‐dependent density functional theory (TDDFT) calculations to correlate the electronic/optical properties of Au_40_(S‐*t*Bu)_24_ with its structure. The structural data from SCXRD was adopted for the simulations. The simulated spectrum (Figure 2a, blue line) shows a prominent peak at 506 nm and two weaker bands beyond 600 nm. The simulated absorption profile matches with experiment except for a small shift. Specifically, the peak ≈506 nm (labelled C) consists of multiple transitions with major ones including HOMO–11 to LUMO+6 (28%), HOMO–12 to LUMO+3 (16%), and HOMO–13 to LUMO+2 (13%) (Figure 2b; Figure S4, Supporting Information), while other transitions with contributions below 10% are identified but not listed here. Similarly, the peak at ≈632 nm (B) comprises the main transitions of HOMO–7 to LUMO, HOMO–8 to LUMO+1, and HOMO–2 to LUMO+3, each with ≈14–17% contributions. The peak at ≈718 nm (A) is predominantly from the HOMO–1 to LUMO+1 transition.

**Figure 3:**
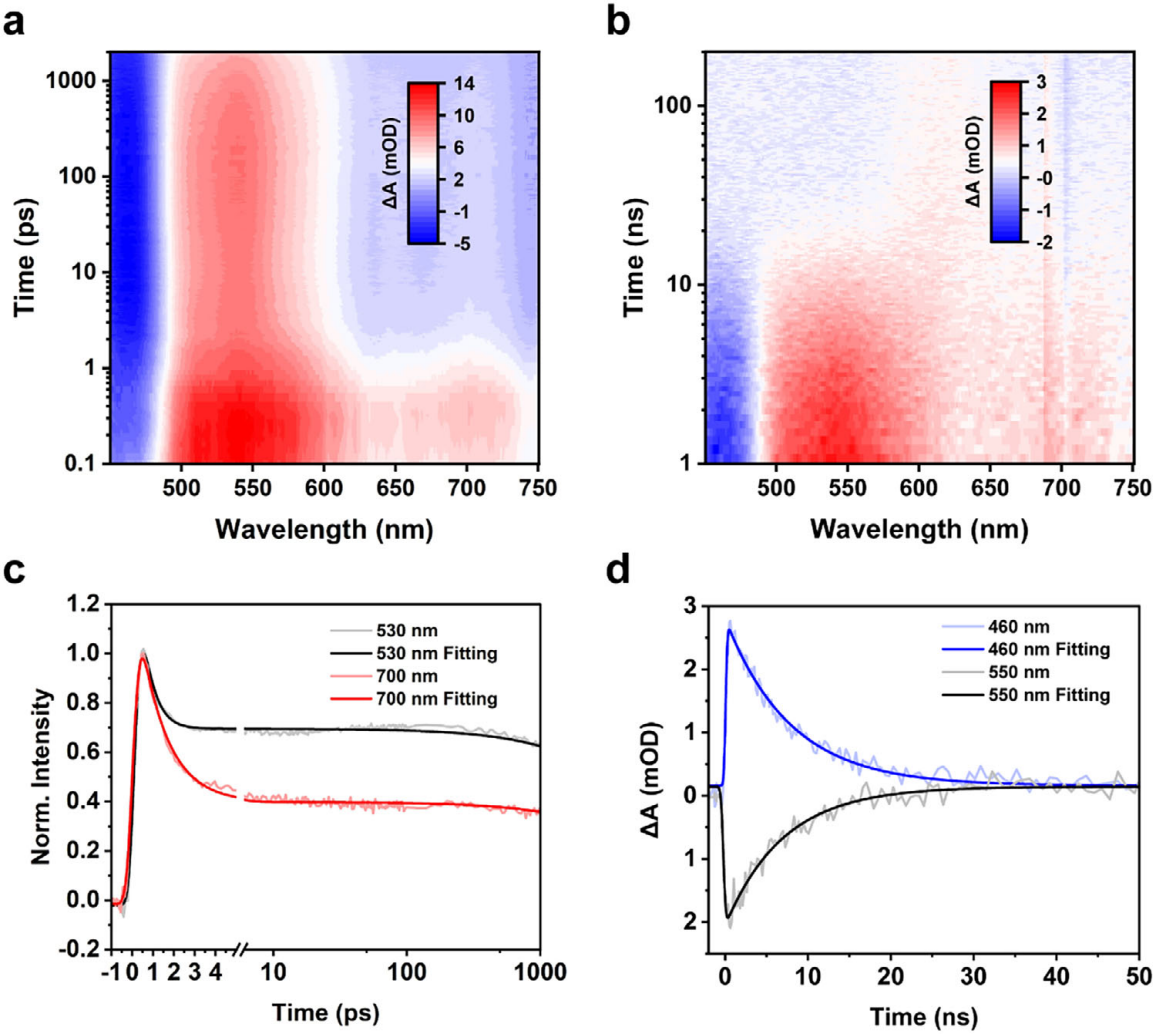
Transient absorption analysis of excited‐state dynamics of Au_40_(S‐*t*Bu)_24_. a) Femtosecond scale fs‐TA data map, b) nanosecond scale ns‐TA data map, c) kinetic traces for fs‐TA measurements, and d) kinetic traces for ns‐TA measurements. All excitation was at 360 nm.

To investigate the electron dynamics of Au_40_(S‐*t*Bu)_24_, femtosecond transient absorption (fs‐TA) and nanosecond transient absorption (ns‐TA) measurements were conducted using a 360 nm excitation wavelength. The fs‐TA spectrum (**Figure** **3**a) reveals a prominent negative feature at 450 nm consistent with the steady‐state absorption spectrum (see Figure 2a above); thus, it corresponds to the ground‐state bleach (GSB). Additionally, a broad positive band spanning 495–750 nm represents the excited‐state absorption (ESA), with two distinct peaks centered at 530 and 570 nm. Kinetic traces extracted at 530 and 700 nm (Figure 3c) reveal an ultrafast decay within the first few picoseconds, indicative of the internal conversion (IC) process from a higher excited state (S_n_) to the lowest singlet state (S_1_). The absence of such a rapid decay in the traces obtained with 750 nm excitation (Figure S5, Supporting Information) further supports the assignment of this process to IC. Global analysis yields two evolution‐associated spectra (EAS), effectively deconvoluting the fs‐TA data (Figure S6, Supporting Information). The first species, **EAS 1**, corresponds to the higher singlet excited state initiated by the pump pulse, which subsequently undergoes an IC process with a time constant of 1.1 ps, leading to the formation of the lowest singlet excited state **EAS 2**, which shows a lifetime of 11.9 ns (not accurate due to the fs‐TA insufficient range). To comprehensively map the excited‐state dynamics, ns‐TA experiments were conducted using 360 nm excitation (Figure 3b), revealing a single‐exponential decay with an accurate lifetime of 7.7 ns (Figure 3d), in agreement with the gross 11.9 ns lifetime obtained from fs‐TA via global analysis (Figure S6, Supporting Information). Notably, ns‐TA measurements with 750 nm excitation exhibited identical dynamics to those observed with 360 nm excitation, further confirming a consistent excited‐state lifetime of 7.7 ns (Figure S6, Supporting Information, panel **d**). This short‐lived lifetime suggests that a significant population of excited species undergoes rapid vibrational relaxation to the S_0_ state. In contrast, the fcc Au_40_(*o*‐MBT)_24_ quasi‐isomer exhibits a long excited state lifetime of 640 ns,^[^ ^28^ ^]^ thus, the non‐compact structure of Au_40_(S‐*t*Bu)_24_ leads to a nearly two orders of magnitude acceleration in electron relaxation owing to much stronger electron‐vibration coupling, which may be promising in terahertz emission.^[^ ^29^ ^]^ To gain insight into the electron‐vibration coupling, we measured the photoluminescence of Au_40_(S‐*t*Bu)_24_, which exhibits weak near‐infrared emission centered at 850 nm (Figure S7, Supporting Information) with a quantum yield of 1%. The low quantum yield is a result of strong nonradiative decay, which indirectly confirms strong electron‐vibration coupling.^[^ ^13^ ^]^

**Figure 4:**
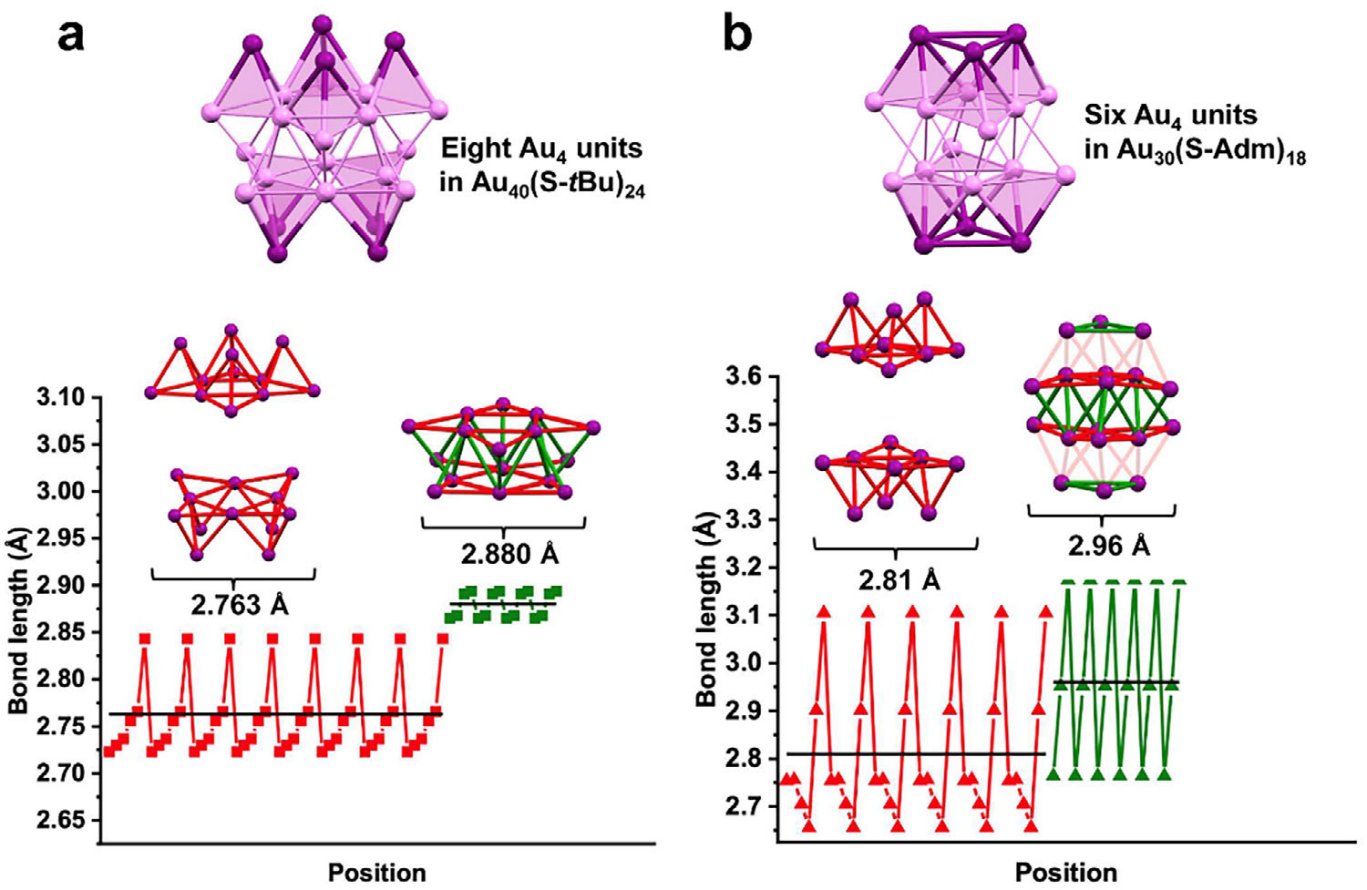
Local tetrahedral Au_4_ units and Au─Au bond length distributions in kernels of a) Au_40_(S‐*t*Bu)_24_ and b) *hcp* Au_30_(S‐Adm)_18_ with comparable photoexcited state lifetimes. Color code: red bonds are within each Au_4_, and green bonds are between Au_4_ units.

The drastic difference in excited‐state lifetime between the two Au_40_ structures is interesting. To gain insight, we carefully analyzed the Au─Au bond lengths in the metal core and identified eight local tetrahedral units (Au_4_), and interestingly, such Au_4_ units form top and bottom square arrays, **Figure** **4**a. Each Au_4_ unit has shorter bonds (average 2.77 Å) than those between neighboring Au_4_ units (average 2.88 Å). This is reminiscent of the triangular arrays of Au_4_ units in hcp Au_30_(SR)_18_ (Figure 4b), which was reported to have a 1 ns excited‐state lifetime.^[^ ^30^ ^]^ Thus, we believe the common feature of cyclic arrays of Au_4_ units should be the reason for the short photoexcited state lifetimes; note: no such feature is found in fcc Au_40_(*o*‐MBT)_24_. Previously, theoretical work on hcp Au_30_(SR)_18_ revealed wavefunction symmetry‐forbidden nature for the radiative decay (i.e., no luminescence) and strong vibrations of surface motifs (i.e., strong nonradiative decay).^[^ ^31^ ^]^ Thus, we rationalize that such effects may also exist in the non‐close packed Au_40_(S‐*t*Bu)_24_ as in hcp Au_30_(SR)_18_ due to their common nature of cyclic arrays of Au_4_ units. Earlier theoretical analysis by Yu et al.^[^ ^32^ ^]^ also identified the critical roles of nonadiabatic coupling and pure dephasing time between the initial and final states in dictating the electron dynamics, that is, the larger nonadiabatic coupling and longer coherence time found in hcp Au_30_(SR)_18_ can be correlated well with the shorter lifetime of Au_30_(SR)_18_ compared to the fcc NCs of similar E_g_.^[^ ^32^ ^]^ Future work will confirm whether the above factors identified in Au_30_(SR)_18_ can be applied to the case of non‐close‐packed Au_40_(S‐*t*Bu)_24_, having similar cyclic arrays of Au_4_ units in the metal core.

## 3 Conclusion

In summary, this work reports a rare crystal structure observed in Au_40_(S‐*t*Bu)_24_, which is in contrast to the fcc Au_40_(*o*‐MBT)_24_. From the application point of view, achieving control of both long and short lifetimes is important, with the long lifetimes being important for photocatalysis,^[^ ^33^, ^34^, ^35^, ^36^ ^]^ and the short ones for single photon emission and biomedical imaging.^[^ ^37^, ^38^, ^39^, ^40^ ^]^ The unique 1D channel that connects the interior and exterior of the Au_40_(S‐*t*Bu)_24_ nanocluster holds promise in future explorations of ion channeling and ion selection, artificial photosynthesis and biocatalysis,^[^ ^41^ ^]^ as well as potential opportunities for new host‐guest chemistry.^[^ ^42^ ^]^

## 4 Experimental Section

The synthesis of Au_40_(S‐*t*Bu)_24_ was carried out in solution by a wet chemistry approach. The target product was isolated by thin layer chromatography. Crystallization was performed by slow diffusion of ethanol (non‐solvent) into a dichloromethane solution of Au_40_(S‐*t*Bu)_24_. Black block‐shaped crystals were obtained after 4 days at room temperature, followed by X‐ray crystallographic analysis. More details and other characterization are provided in the Supporting Information.

## Conflict of Interest

The authors declare no conflict of interest.

## Supporting information



Supporting Information

## Data Availability

The data that support the findings of this study are available in the supplementary material of this article.
