# Supplementary material for: Phase Engineering of Nanogold: Non‐Close Packed Square Planes in A′B′ Stacking with a 0.5 Å Channel
Source: Adv Mater. 2025 Dec 16;38(8):e17188. doi: 10.1002/adma.202517188 (PMC12878809; doi:10.1002/adma.202517188)
Supplement: Supplementary file 1 — Supporting Information [file ADMA-38-e17188-s001.pdf]

ADVANCED MATERIALS

Supporting Information

for *Adv. Mater.*, DOI 10.1002/adma.202517188

Phase Engineering of Nanogold: Non-Close Packed Square Planes in A′B′ Stacking with a 0.5 Å Channel

*Yitong Wang, Zhongyu Liu, Christopher G. Gianopoulos, Wei Zhang, Dominic Alfonso, Guiying He, Kristin Kirschbaum, Meng Zhou and Rongchao Jin**

(Supporting Information)

Phase Engineering of Nanogold: Non-Close Packed Square Planes in A′B′ Stacking with a 0.5 Å Channel

Yitong Wang[†], Zhongyu Liu[†], Christopher G. Gianopoulos[‡], Wei Zhang[§], Dominic Alfonso[‖], Guiying He,[†] Kristin Kirschbaum[‡], Meng Zhou[§], and Rongchao Jin[†*]

[†]Department of Chemistry, Carnegie Mellon University, Pittsburgh, Pennsylvania 15213, United States
[‡]Department of Chemistry and Biochemistry, University of Toledo, Toledo, Ohio 43606, United States
[§]Hefei National Research Center for Physical Sciences at the Microscale, Department of Chemical Physics, University of Science and Technology of China, Hefei, Anhui 230026, China
[‖]National Energy Technology Laboratory, United States Department of Energy, Pittsburgh, Pennsylvania 15236, United States

[*]To whom correspondence should be addressed: rongchao@andrew.cmu.edu

Experimental

Chemicals. Tetrachloroauric (III) acid ($HAuCl_4 \cdot 3H_2O$, 99.99% metal basis, Aldrich), tert-butyl thiol (S-tBu, 98%, Aldrich), tetrahydrofuran (HPLC grade, ≥99.9%, Aldrich), toluene (HPLC grade, ≥99.9%, Aldrich), dichloromethane (DCM, ACS reagent, ≥99.5%, Aldrich), acetonitrile (HPLC grade, ≥99.9%, Aldrich), methanol (MeOH, HPLC grade, ≥99.9 %, Aldrich), ethanol (EtOH, HPLC grade, ≥99.9 %, Aldrich). All chemicals were used as received without further purification. Deionized water was prepared with a Barnstead NANOpure Diamond system (18.2 MΩ cm). Thin-layer chromatography (TLC) plates were purchased from iChromatography (silica gel, 250 µm).

Synthesis of Au_{40}(S-*t*Bu)$_{24}$

89 mg of $HAuCl_4 \cdot 3H_2O$ and 65.5 µL tert-butyl thiol were dissolved in 15 ml of THF under rapid stirring (~500 rpm). The solution turned to deep orange in 30 min. Then, a freshly prepared $NaBH_4$ solution (38 mg, 1 mmol) was added to the reaction mixture in a dropwise manner. Alternatively, a freshly prepared methanol solution of 350 mg $(CH_3)_3CNH_2 \cdot BH_3$ was added all at once. The reaction was allowed to proceed for 8 hr at room temperature. After the reaction, the solvent was rotary evaporated, giving rise to a dark oil-like liquid, which was then precipitated with methanol. The precipitate was washed by excess methanol, and the obtained black product was further purified by thin layer chromatography (TLC) with a developing solvent of hexane : DCM = 3:2 (v/v). Crystallization of Au_{40}(S-*t*Bu)$_{24}$ was carried out by liquid phase diffusion of ethanol (3 mL) into a dichloromethane solution of Au_{40}(S-*t*Bu)$_{24}$ (1 mL, saturated) at room temperature. Black block shaped crystals were obtained after 4 days.

X-ray Crystallography Analysis of Au_{40}(S-*t*Bu)$_{24}$ Crystals

A black block shaped crystal of Au_{40}(S-*t*Bu)$_{24}$ (see photo below) was used for data collection on a Bruker Duo diffractometer with a PHOTON II detector and IµS CuKα radiation (1.54178 Å) at 200 K. The structure was solved in the tetragonal space group P42(1)2. Integration and scaling of

the data yielded 168,677 reflections, of which 9,215 were unique (and 7,449 unique data with $I > 2\sigma(I)$), to a maximum of $\theta = 70.68°$ (d = 0.82 Å) with a completeness of 98.4% (99.8% to $\theta = 67.68°$; d = 0.83 Å); and an R_{int} of 18.71%.

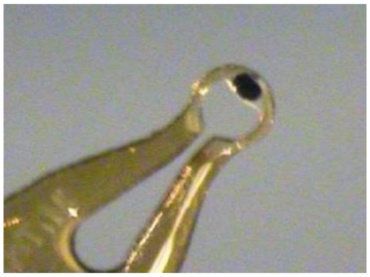

A reasonable quality three-component twinned crystal was found after screening of crystals. Data were integrated as a three-component twin, with twin fractions 0.49, 0.24 and 0.27. For the final refinement, only the non-overlapping reflections from all three components were used. All Au, S and C atoms were refined with anisotropic thermal displacement parameters with carbon atoms subjected to the rigid bond restraint (RIGU). Hydrogen atoms were placed in idealized positions and treated with a riding model. High residual density maxima were found in the vicinity of the Au and S atoms, which could be modeled as a small disorder component of all Au and S atoms (78% and 22%). Disordered C atoms belonging to the minor component could not be modeled. In the final model, the tBu groups were constrained to 78% occupancy in agreement with the occupancy of the Au and S atoms of the major component. Thus, the final model consisted of 506 parameters with 108 restraints.

The final refinement on F^2 converged at $R_1 = 6.44\%$ ($I > 2\sigma(I)$) and $wR_2 = 18.24\%$ (all data). The goodness-of-fit was 1.165. The largest residual density extrema were 2.567 and –1.106 ($e^-/Å^3$) with an RMS deviation of 0.533 $e^-/Å^3$.

Steady-State UV-Vis-NIR Measurements

UV-Vis-NIR spectra of Au nanoclusters were collected with a UV-3600 Plus UV-VIS-NIR spectrophotometer (Shimadzu).

Ultrafast Spectroscopic Measurements

The fs-TA spectra were measured using a home-built femtosecond pump-probe set-up. The laser pulse (800 nm, 35 fs pulse width, 1 kHz repetition rate) was generated by a regeneratively amplified Ti:Sapphire laser (Coherent Astrella-USP, USA). The output of the pulse is then divided into two beams with a beam splitter. The probe beam was delayed with a computer-controlled optical delay line and then focused onto a thin sapphire plate to generate a white light supercontinuum which was split into two beams by using a broadband 50/50 beam splitter as the signal and reference beams. The TOPAS Prime (Light Conversion) was used to generate the pump pulses with central wavelength of 360 nm (0.12 μJ) and 750 nm (0.1 μJ), respectively. The focused pump and probe pulses were overlapped in a sample cuvette. The mutual polarization between the pump and probe beams was set to the magic angle (54.7°) by placing a half-wave plate in the pump beam.

The ns-TA spectra were measured by a commercial spectrometer (Time-Tech Spectra). The generation of the pump beam is the same as that in the fs-TA (see above). The probe beam was generated from a supercontinuum laser (LEUKOS-DISCO, French) with a spectral region from 350 to 1800 nm, the repetition rate is 2 kHz, pulse width is 700 ps - 1 ns.

No photodegradation was found after fs- and ns-TA experiments by checking the steady-state absorption spectra.

DFT Calculations of Electronic Structure and UV-Vis Absorption Spectrum

We first generated the $Au_{40}(SH)_{24}$ model by obtaining atomic coordinates from the experimentally-solved Au_{40}(S-*t*Bu)$_{24}$ crystal structure. The organic fragment of the ligands was modeled using a -H moiety to generate a computationally tractable model while accurately capturing the geometrical structure of the nanocluster. The geometry was then preoptimized using plane-wave density functional theory (DFT) as implemented in the Vienna Ab Initio Simulation Package Package (VASP) version 5.4.4.1 Perdew-Burke-Ernzerhof (PBE) functional, projector augmented wave (PAW) pseudopotential, and plane-wave basis sets with cutoff energy of 520 eV were employed. A three-dimensional 30 Å × 30 Å × 30 Å periodic box was inserted in the 84-atom $Au_{40}(SH)_{24}$ models to exclude artificial periodic interaction. The sampling of the Brillouin zone was conducted with a Γ-point k-point mesh, and the ionic and electronic convergence limit was set to 0.03 eV/Å and 1×10-5 eV, respectively. The Methfessel-Paxton scheme was utilized with a modest smearing width of 0.2 eV, and the total energies were extrapolated to $\sigma \rightarrow 0.2$.

These structures serve as reference points for subsequent calculation of the photo-absorption and Raman spectra using protocols implemented in TURBMOLE package (version 7.4).[3] Geometry reoptimization without symmetry restriction was first conducted prior to the simulation. The PBE functional, def2-SV(P) basis set [4], effective core potentials to describe the inner electrons, and m4 quality quadrature grids[5] were chosen. The multipole-accelerated resolution of identity MARI-J is used for speedup.[6] For photo-absorption simulation, enhanced reliability and accuracy of excitation energetics was aspired through the deployment of time-dependent density functional theory (TD-DFT). The 700 lowest singlet-to-singlet vertical excitation energies were determined within the resolution of identities approximation.[7] Simulated spectra were generated by convolving the calculated absorption energies and intensities with a Gaussian function of sigma set to 40 nm, and sampling the energy over 700 points within a 300-900 nm range. The harmonic vibrational Raman spectra were also simulated using TURBOMOL 7.4 at DFT-PBE/ def2-SV(P)/MARI-J level. A normal mode analysis was applied to the $Au_{40}(SR)_{24}$ ground state geometries to ensure local minima. The bare TURBOMOL derivative of anisotropic polarizability output in atomic units ($a_o^2 m_e^{-0.5}$) was plotted versus the wavenumber (cm^{-1}). Calculated spectra are shown as Gaussian-convoluted lines with σ broadening of 0.1 cm^{-1}.

[1] J. P. Perdew, K. Burke and M. Enzerhof, *Phys. Rev. Lett.*, 1996, **77**, 3865-3868; G. Kresse and D. Joubert, *Phys. Rev. B*, 1999, **59**, 1758-1775; G. Kresse and J. Furthmüller, *Comp. Mat. Sci.*, 1996, **6**, 15-50.

[2] M. Methfessel and A. T. Paxton, *Phys. Rev. B*, 1989, **40**, 3616-3621.

[3] R. Ahlrichs, M. Bär, M. Häser, H. Horn, C. Kölmel, *Chem. Phys. Lett.* **1989**, *162*, 165-169.

[4] F. Weigend, M. Häser, H. Patzelt, R. Ahlrichs, *Chem. Phys. Lett.* **1998**, *294*, 143-152

[5] O. Treutler and R. Ahlrichs, J. Chem. Phys. **102**, 346 (1995).

[6] M. Sierka, A. Hogekamp, R. Ahlrichs, J. Chem. Phys. **118**, 9136-9148 (2003).

[7] F. Weigend, M. Häser, *Theor. Chem. Acc.* **1997**, *97*, 331-340.

Supporting Figures S1-S7:

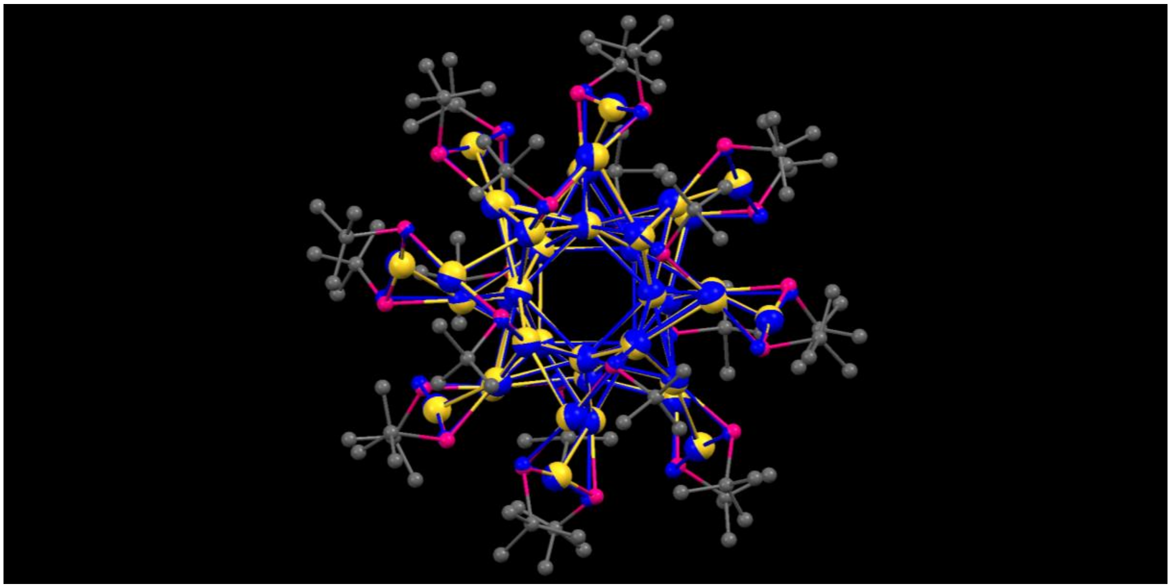

Figure S1. An overlap view of the two configurations (75% vs 25%) of Au_{40}(S-*t*Bu)$_{24}$. No noteworthy difference is identified.

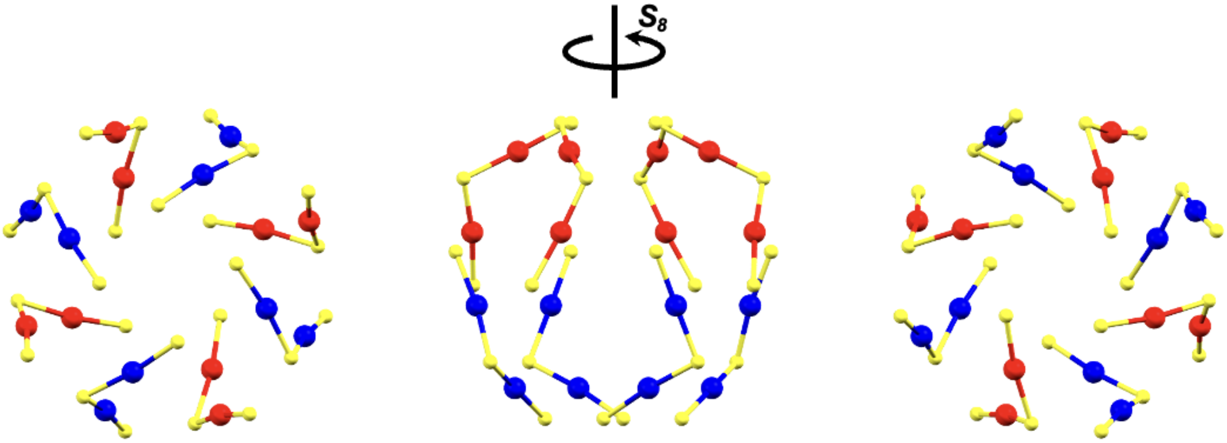

Figure S2. The patterns of surface motifs of Au_{40}(S-*t*Bu)$_{24}$ with –R groups omitted for clarity. Color code: blue/red = gold; yellow = sulfur.

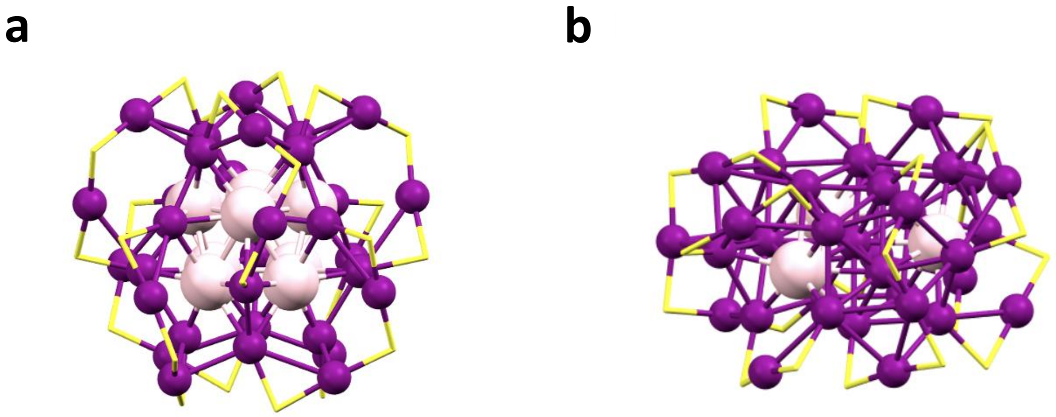

Figure S3. The uncoordinated Au atoms (in pink color) of the two Au_{40} NCs. (a) New structure of Au_{40}(S-*t*Bu)$_{24}$, (b) Case of fcc Au_{40}(*o*-MBT)$_{24}$, where *o*-MBT stands for *ortho*-methylbenzenethiolate.

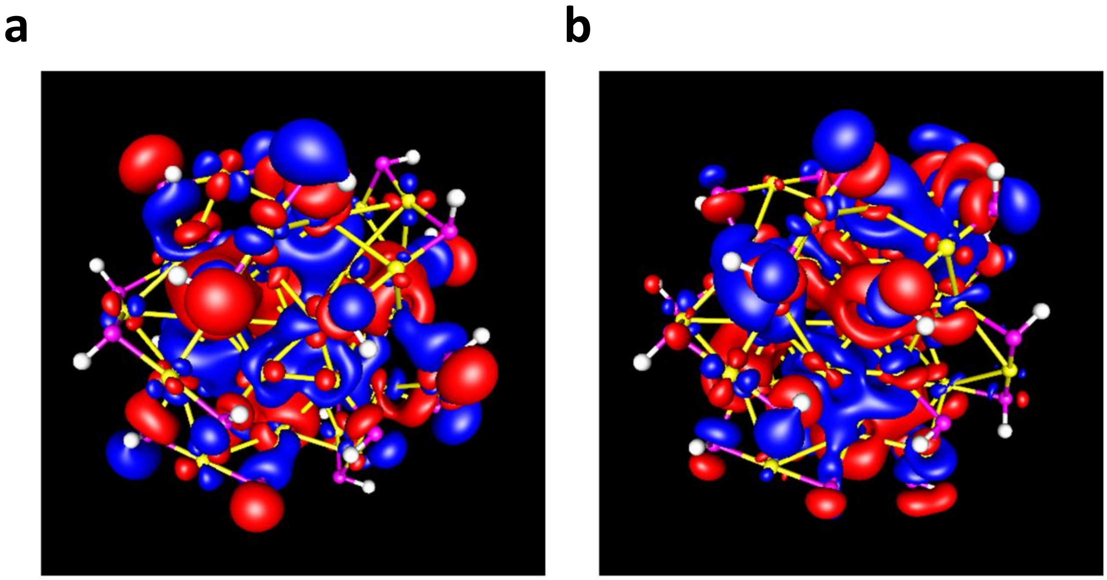

Figure S4. DFT-simulated frontier orbitals of the Au_{40}(S-*t*Bu)$_{24}$ nanocluster. (a) HOMO distribution, and (b) LUMO distribution.

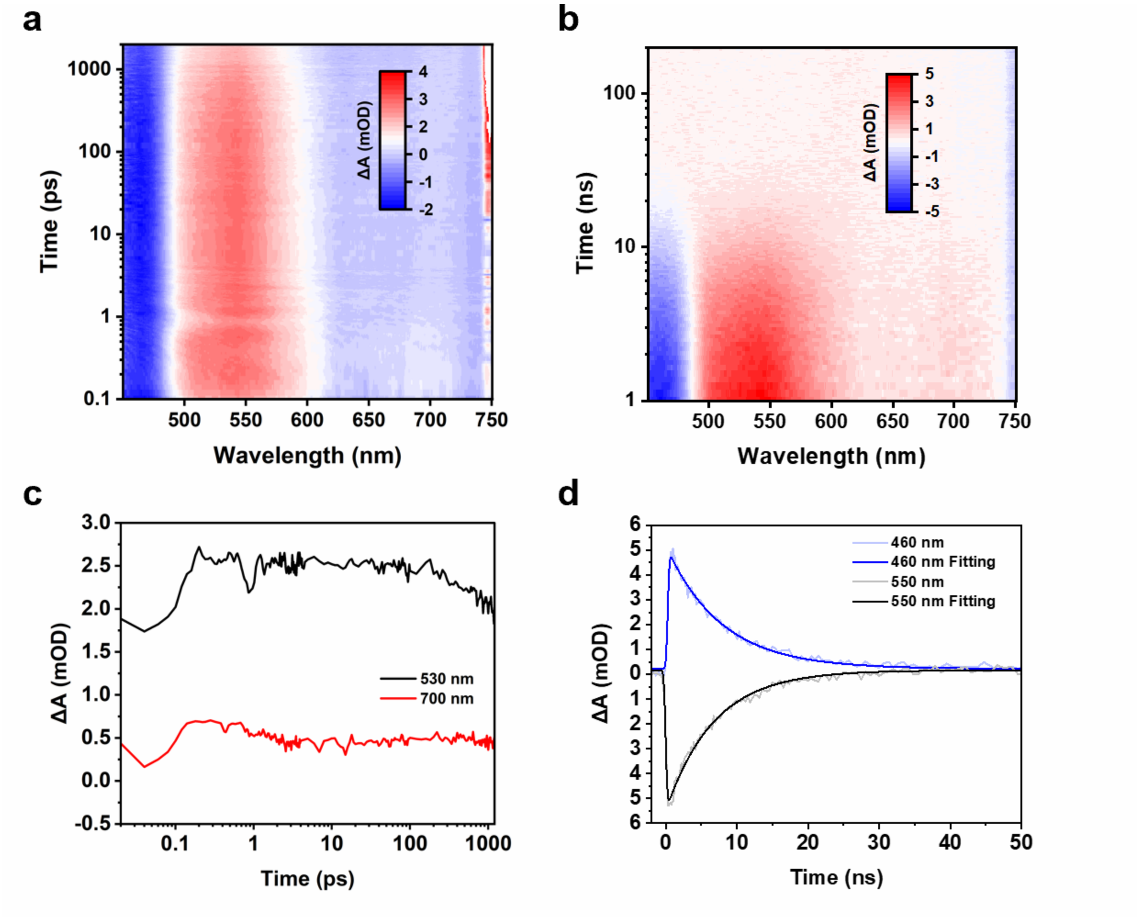

Figure S5. Electron dynamics of $Au_{40}(S\text{-}tBu)_{24}$. (a) fs-TA data map at 750 nm excitation, (b) ns-TA data map under 750 nm excitation, (c) and (d) the kinetic traces at different wavelengths for the fs and ns ranges.

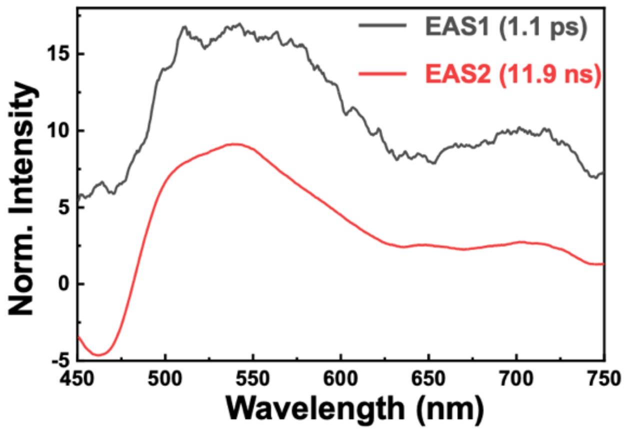

Figure S6. The global analysis results of the fs-TA data of $Au_{40}(S\text{-}tBu)_{24}$. Note that the 11.9 ns value is less accurate since fs-TA has an insufficient range. The ns-TA gives a 7.7 ns lifetime (accurate).

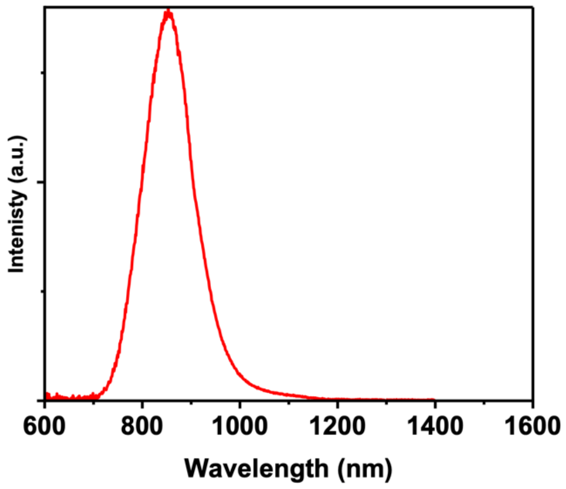

Figure S7. The photoluminescence spectrum of $Au_{40}(S\text{-}tBu)_{24}$ in toluene (excitation: 365 nm), quantum yield ~1%.

Table S1. Sample and crystal data for $Au_{40}(S\text{-}t\text{Bu})_{24}$.

Chemical formula	$C_{96}H_{216}Au_{40}S_{24}$	
Formula weight	10018.94 g/mol	
Temperature	200(2) K	
Wavelength	1.54178 Å	
Crystal system	Tetragonal	
Space group	$P42_12$	
Unit cell dimensions	a = 18.2082(5) Å	α = 90°
	b = 18.2082(5) Å	β = 90°
	c = 29.5513(13) Å	γ = 90°
Volume	9797.4(7) $Å^3$	
Z	2	
Density (calculated)	3.396 g/cm^3	
Absorption coefficient	57.241 mm^{-1}	
F(000)	8672.0	
Theta max	67.473°	
h, k, l max	21, 21, 35	
R (reflections)	0.0692 (7263)	
wR (reflections)	0.1970 (8833)	
Coverage of independent reflections	99.5%	
Absorption correction	Numerical Mu From Formula	
Max. and min. transmission	0.1410 and 0.0320	
Refinement method	Full-matrix least-squares on F^2	
Refinement program	SHELXL-2019/1 (Sheldrick, 2019)	
Function minimized	$\Sigma w(F_o^2 - F_c^2)^2$	
Data / restraints / parameters	31983 / 269 / 1301	
Goodness-of-fit on F^2	1.165	
Final R indices[$I>2\sigma(I)$]	R1 = 6.44%, wR2 = 18.24%	
Largest diff. peak and hole	2.567 and -1.106 $eÅ^{-3}$	
R.M.S. deviation from mean	0.533 $eÅ^{-3}$	
